# Size differences of Arctic marine protists between two climate periods—using the paleoecological record to assess the importance of within‐species trait variation

**DOI:** 10.1002/ece3.2592

**Published:** 2016-11-27

**Authors:** Erik A. Mousing, Sofia Ribeiro, Chelsea Chisholm, Antoon Kuijpers, Matthias Moros, Marianne Ellegaard

**Affiliations:** ^1^ Center for Macroecology, Evolution and ClimateNatural History Museum of DenmarkUniversity of CopenhagenCopenhagenDenmark; ^2^ Department of Glaciology and ClimateGeological Survey of Denmark and Greenland (GEUS)CopenhagenDenmark; ^3^ Leibniz Institute for Baltic Sea Research Warnemünde (IOW)WarnemündeGermany; ^4^ Department of Plant and Environmental SciencesUniversity of CopenhagenCopenhagenDenmark

**Keywords:** cell size, dinoflagellate cysts, interspecific, intraspecific, paleoecology, temperature

## Abstract

Mean body size decreases with increasing temperature in a variety of organisms. This size–temperature relationship has generally been tested through space but rarely through time. We analyzed the sedimentary archive of dinoflagellate cysts in a sediment record taken from the West Greenland shelf and show that mean cell size decreased at both intra‐ and interspecific scales in a period of relatively warm temperatures, compared with a period of relatively cold temperatures. We further show that intraspecific changes accounted for more than 70% of the change in community mean size, whereas shifts in species composition only accounted for about 30% of the observed change. Literature values on size ranges and midpoints for individual taxa were in several cases not representative for the measured sizes, although changes in community mean size, calculated from literature values, did capture the direction of change. While the results show that intraspecific variation is necessary to accurately estimate the magnitude of change in protist community mean size, it may be possible to investigate general patterns, that is relative size differences, using interspecific‐level estimates.

## 1 Introduction

Determining the impact of temperature increase on the Earth's biota is an important aspect of global change research. Studies have shown that mean body size decreases with increasing temperature in a variety of organisms (Sheridan & Bickford, 2011). This pattern has also been observed for protists (Atkinson, Ciotti, & Montagnes, 2003; Ellegaard, Lewis, & Harding, 2002; Peter & Sommer, 2012; Yvon‐Durocher, Montoya, Trimmer, & Woodward, 2011), which are single‐celled eukaryotes, that form the basis of aquatic food webs. Future changes in their size composition potentially impacts ecosystem structure and functioning by affecting the subsequent trophic layers and biogeochemical cycling (Finkel et al., 2010; Hilligsøe et al., 2011; Kiørboe, 1993). Regional‐scale empirical studies where protist size and temperature have been investigated on large spatial gradients show the same relationship (López‐Urrutia & Morán, 2015; Mousing, Ellegaard, & Richardson, 2014) and this relationship has been used to predict an increase in the relative importance of small cells in aquatic environments in a future warmer climate (Daufresne, Lengfellner, & Sommer, 2009; Morán, López‐Urrutia, Calvo‐Díaz, & Li, 2010; Winder, Reuter, & Schladow, 2009).

The method of predicting temporal changes based on contemporary spatial gradients is called space‐for‐time substitution (Pickett, 1989). This method has been widely used within various fields because it allows investigating patterns and processes that are otherwise hard or impossible to observe (Blois, Williams, Fitzpatrick, Jackson, & Ferrier, 2013). In ecology, it has been used to predict future and past species distributions (i.e., species distribution modeling) and diversity patterns for many different organism groups (e.g., Barton, Irwin, Finkel, & Stock, 2016; Currie, 2001; Eskildsen et al., 2013). Even though space‐for‐time substitution has been shown to be reliable in some cases (e.g. Blois et al., 2013), the predictions derived from the method are challenged by the fact that it is usually not possible to validate them. For protists, however, it is actually possible to directly investigate changes in size structure through time by investigating remains preserved in the sedimentary record; that is, the paleoecological approach. By comparing past patterns in protist community composition to historical and/or reconstructed changes in climate (e.g., temperature), it is possible to investigate how community size structure has changed under past environmental shifts and thus directly assess whether protists were indeed smaller in relatively warm periods compared to relatively cold periods.

Here, we focus on dinoflagellates which constitute a major part of protist communities in aquatic ecosystems and include a large diversity of feeding strategies, ranging from autotrophic to heterotrophic species (many are mixotrophic). Some dinoflagellates (thus far documented for about 20%) produce a resting stage, called a cyst, as part of their life cycle (Head, 1996). These cysts are nonmotile and sink to the seafloor where they accumulate and may retain their capacity for germination for at least a century (Ribeiro et al., 2011). Dinoflagellate cysts are preserved in the sediment for up to millions of years and are studied for numerous reasons, including biostratigraphy and paleoenvironmental reconstructions (Dale, 2001). The size of dinoflagellate cysts has been shown to be 1:1 linearly correlated with the size of the motile cells (Finkel et al., 2007) and cyst size can therefore be considered a suitable proxy for the size of actively dividing dinoflagellates in the water column prior to encystment.

A few studies have pointed to the great potential of using a paleoecological approach to understand the effects of climate change on protist community size structure (Chen, Irwin, & Finkel, 2011; Finkel, Katz, Wright, Schofield, & Falkowski, 2005; Finkel et al., 2007). However, in most of these paleoecological investigations, protist size and also the community size structure have not been based on measurements of taxa within the actual sediment core. Instead, individual taxa found in the sediment core have been assigned fixed size values which have been found in the scientific literature. Using these taxon‐specific size values, changes in community size structure have then been calculated based on changes in community taxonomic composition (i.e., interspecific change).

Literature values have also been used prolifically as species means for other organism groups, including plants (Adler et al., 2014; Cornwell & Ackerly, 2009), fish (Stuart‐Smith et al., 2013) and mammals (Whitmee & Orme, 2012). However, a number of reviews in the higher plant literature have pointed to the importance of intraspecific variation (Bolnick et al., 2011; Violle et al., 2012). Explorations of the contribution of intraspecific variation to community responses across gradients have demonstrated that community patterns may be misinterpreted if solely examined at the species level (Carlucci, Debastiani, Pillar, & Duarte, 2015; Jung, Violle, Mondy, Hoffmann, & Muller, 2010). Within‐species variation may even be more important depending on the scale of the analysis, which has generally been tested through space but not time. This emphasizes the importance of considering the implications of using species’ means as adequate descriptors of an individual's trait expression.

In this study, we use paleoecological analyses to assess shifts in protist community size structure with past temperature changes by analyzing size distributions of dinoflagellate cysts in a marine sediment core collected in the Disko Bay, off West Greenland. Disko Bay is an area of interest with regard to global change because it is under the influence of the Jakobshavn Isbræ glacier, the largest and fastest ice stream in the Northern Hemisphere (Joughin, Smith, Shean, & Floricioiu, 2014). This region currently drains 7% of the Greenland Ice Sheet (Roberts & Long, 2005), contributing significantly to sea level rise (Howat et al., 2011), and is particularly sensitive to future warming (Nick et al., 2013).

We sought to answer the questions: Are protists significantly smaller during a relatively warm period compared to a relatively cold period? and, what is the relative importance of intraspecific and interspecific cell size changes in facilitating a change in the community mean size?. In addition, by comparing measured sizes of dinoflagellate cysts to available literature values, we sought to answer the question: Are literature values of species size adequate to describe local species size distributions and changes in community mean size?

## 2 Methods

### 2.1 Core collection and dating

**Figure 1:**
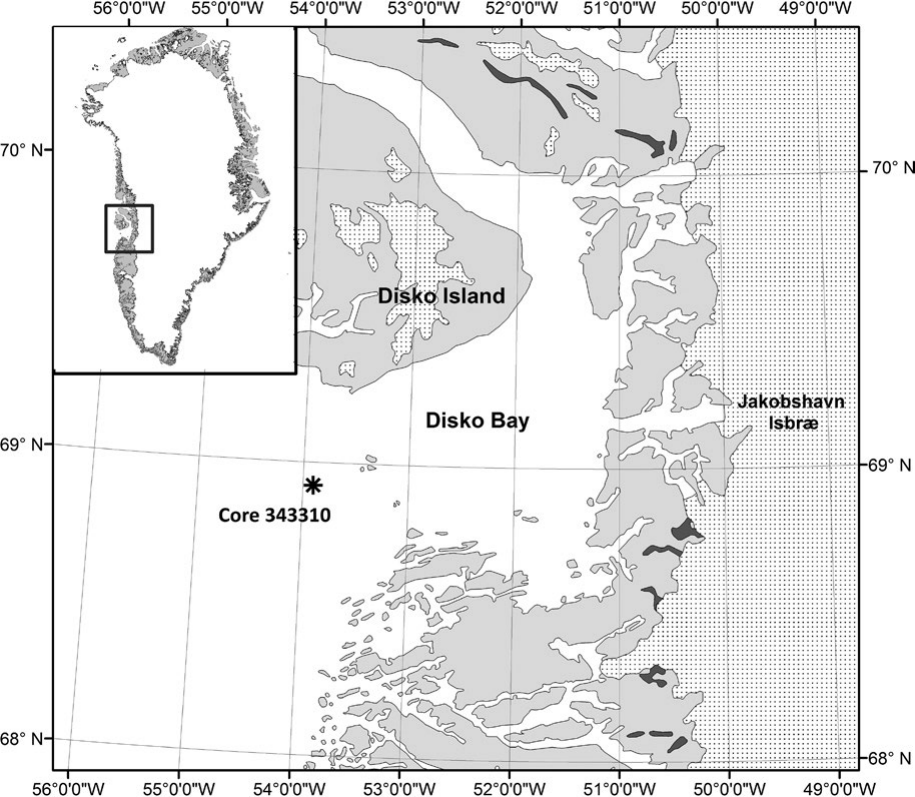
Map of the Disko Bay area, Greenland and coring site (*)

The samples used in this study originate from a gravity sediment core (343310) retrieved in southwest Disko Bay, a large embayment on West Greenland, at coordinates 68°38′861″ N and 53°49′493″ W and at 855 m water depth (Figure 1) (Harff et al., 2007; Perner et al., 2011; Ribeiro, Moros, Ellegaard, & Kuijpers, 2012). The core had a total length of 940 cm, and a robust chronology was constructed for the entire record on the basis of 20 Accelerator Mass Spectrometry (AMS) ^14^C dates (Perner et al., 2011; Ribeiro et al., 2012).

**Figure 2:**
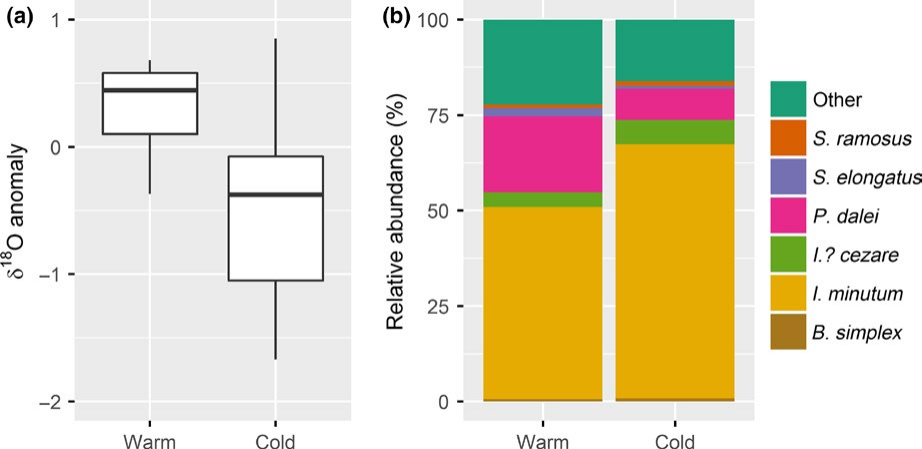
Boxplots showing the median winter δ^18^O temperature anomalies for the warm and cold periods (data from Vinther et al., 2010) (a). The relative distribution of dominant dinoflagellate species for the warm (based on 613 observations) and cold (based on 680 observations) periods (data from Ribeiro et al., 2012) (b) “Other” includes rare species and species unidentifiable to species level (mainly *Brigantedinium* spp.)

For this study, we selected core age‐depths representing two contrasting climate periods. To represent a relatively cold period, we choose 60–61 cm and 80–81 cm core‐depth which, based on radiocarbon dating, falls within about 1,590–1,710 AD (the so‐called Little Ice Age). To represent a relatively warmer period, we chose 140–141 cm and 150–151 cm core‐depth which falls within about 1,270–1,460 AD. These samples were chosen based on the independent ice‐core temperature anomalies reported in Vinther et al. (2010) (Figure 2a) as well as on reconstructed sea ice cover trends (Ribeiro et al., 2012). The methodology for sample processing as well as a paleoclimatic interpretation of the changes in community composition during the last 1,500 years, corresponding to the first 400 cm of the record, is described in detail in Ribeiro et al. (2012).

### 2.2 Morphometric analyses

After treatment of the sediment for removal of the mineral fraction (following a standard palynological protocol with cold 70% HCl and cold 40% HF), the remaining organic fraction between 10 and 150 μm was mixed with glycerin jelly and mounted on permanent microscope slides. For morphometric analyses, slides were examined using an Olympus BH‐2 light microscope at 400× magnification and micrographs were taken of all measured dinoflagellate cysts using an Olympus DP72 digital camera. Measurements were taken using the software Cell F version 2.4. The diameter (measured from the inside of the cyst wall) was determined for approximately 20 cysts of each taxon per sample, amounting to a total of 448 cysts measured.

**Figure 3:**
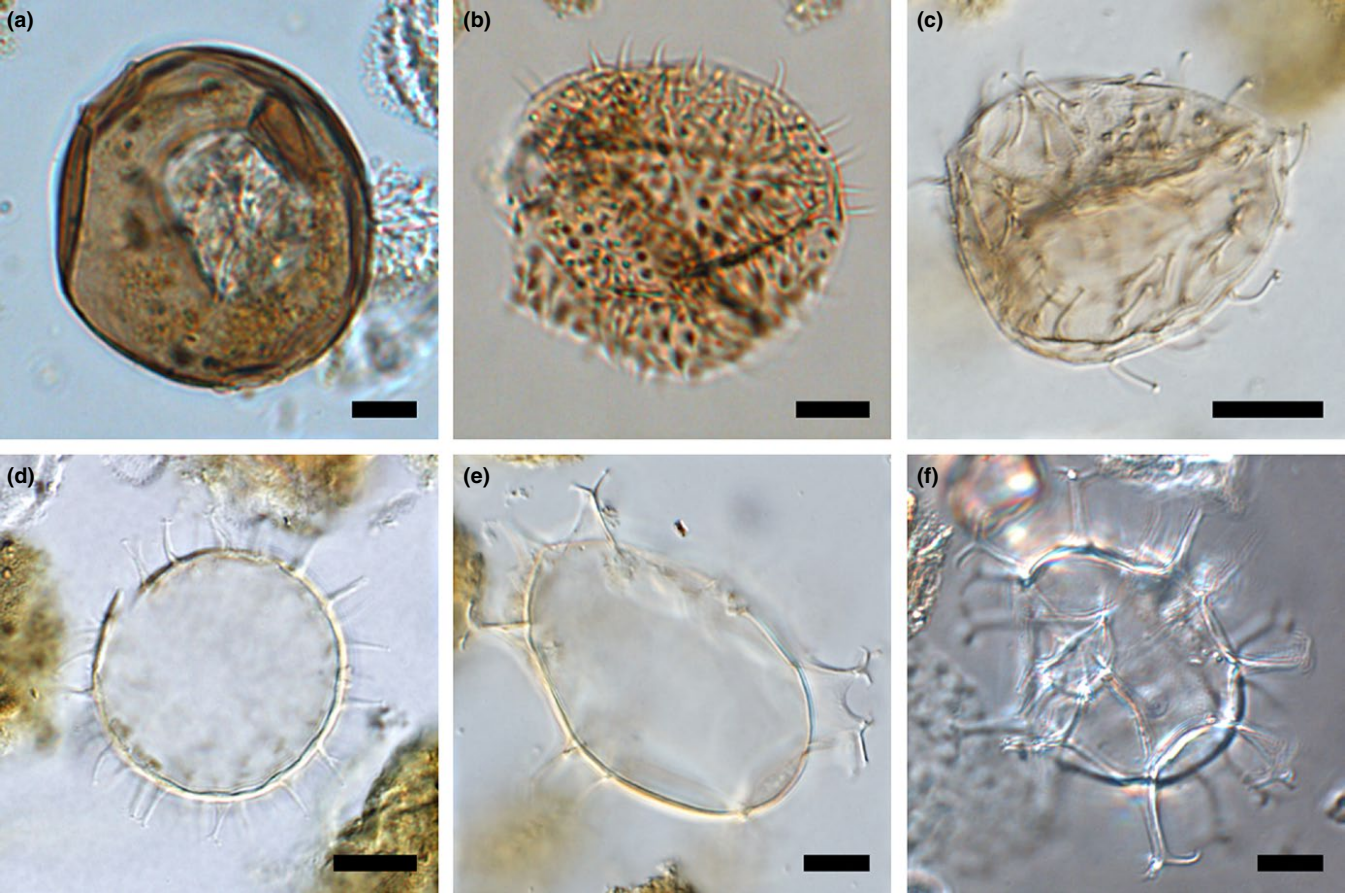
Micrographs of the most abundant species in the studied sediment record: *Brigantedinium simplex* (a), *Islandinium minutum* (b), *Islandinium? cezare* (c), *Pentapharsodinium dalei* (d), *Spiniferites elongatus* (e), and *Spiniferites ramosus* (f). Micrographs were taken using a light microscope with differential interference contrast. Black bars represent 10 μm

In this study, we analyzed the size distribution of the six most abundant identifiable dinoflagellate cyst species in the sediment record from Disko Bay (Figures 2b and 3). Three species of heterotrophic dinoflagellates: *Brigantedinium simplex* (Figure 3a), *Islandinium minutum* (Figure 3b) and *Islandinium? cezare* (Figure 3c); and three species of phototrophic dinoflagellates: *Pentapharsodinium dalei* (Figure 3d), *Spiniferites elongatus* (Figure 3e), and *Spiniferites ramosus* (Figure 3f). Altogether, these six species accounted for ca. 80% of the total dinoflagellate cyst community throughout the record and although 29 taxa were identified in total, most were rare (Ribeiro et al., 2012). In the sediment samples examined in this study, cysts of the species not included were either unidentifiable to species level (mainly *Brigantedinium spp*.) or very rare (constituting <1.5% of the assemblage in total).

For the approximately circular species *B. simplex*,* I. minutum*, and *P. dalei*, the diameter was measured directly when cysts were relatively undamaged and estimated as the average of cyst length and width when cysts were compressed. For the small species *I.? cezare*, cysts were often compressed and therefore the cyst size was measured consistently using the largest diameter measurable. For the *Spiniferites* cysts, *S. ramosus* and *S. elongatus*, the diameter was estimated as the average of cyst length and width.

### 2.3 Numerical analyses

All statistical analyses were performed using R version 3.2.2 (R Core Team, 2015). For data preparation, we used the packages “plyr” version 1.8.3 (Wickham, 2011) and “reshape2” version 1.4.1 (Wickham, 2007). Weighted statistics were performed using the packages “SDMTools” version 1.1–211 (VanDerWal, Falconi, Januchowski, Shoo, & Storlie, 2014) and “weights” version 0.85 (Pasek & Tahk, 2016). Plotting was performed using the packages “ggplot2” version 1.0.1 (Wickham, 2009) and “gridExtra” version 2.0.0 (Auguie, 2015).

**Figure 4:**
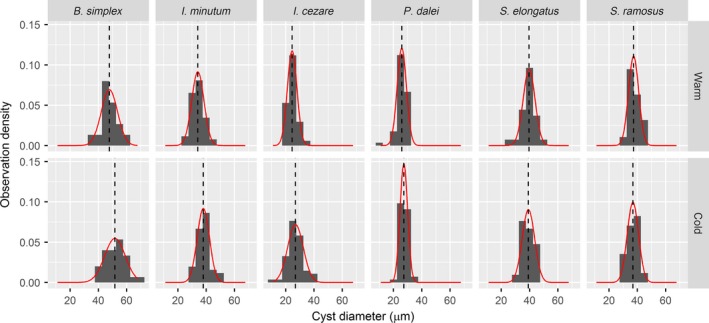
Histograms of the observed distribution of diameter observations for all species in the warm and cold periods. The dashed line is the mean diameter, and the red line is the theoretical normal distribution

For each species, the mean, standard deviation, and standard error of cyst size were calculated and the difference in the mean between the warm and cold periods was tested using a Welch two‐sample *t*‐test for unequal variances (Welch, 1947). Normality was assessed visually by plotting histograms of the data distribution of each species (Figure 4).

Changes in community mean size can be caused by both changes in community structure, that is, species shifts (interspecific changes), and changes in the cyst size of individual species, that is, phenotypic plasticity (intraspecific changes). In order to assess the relative importance of intraspecific vs. interspecific changes, we first calculated the weighted community mean size including the influence of both intra‐ and interspecific changes (*x*_intra+inter_) for each period following Equation 1:

(1) $${\overset{¯}{x}}_{\mathrm{intra}+\mathrm{inter}}=\frac{\sum (\emptyset_{\mathrm{obs}}\times w_{\mathrm{obs}})}{\sum (w_{\mathrm{obs}})}$$

where Ø_obs_ are individual observations of cyst diameter and *w*_obs_ is the weight of each observation given by Equation 2.

(2) $$w_{\mathrm{obs}}=\frac{w_{\mathrm{spe}}}{N_{\mathrm{spe}}}$$

where *N*_spe_ is the number of observations of each species and *w*_spe_ is the weight of each species given by Equation 3.

(3) $$w_{\mathrm{spe}}=\frac{N_{\mathrm{spe}}}{N}$$

where *N*_spe_ is the number of cysts of each species and *N* is the total number of cysts (as in Figure 2b).

We then calculated the weighted community mean size excluding intraspecific changes (*x*_inter_) for each period following Equation 4.

(4) $${\overset{¯}{x}}_{\mathrm{inter}}=\frac{\sum (\emptyset_{\mathrm{avg}}\times w_{\mathrm{spe}})}{\sum (w_{\mathrm{spe}})}$$

where Ø_avg_ is the mean cyst size for each species across both periods and *w*_spe_ is the weight of each species in each period. The relative contribution of intra‐ and interspecific changes to total change can then be calculated following Equations (5), (6), (7).

(5) $$\Delta {\overset{¯}{x}}_{\mathrm{intra}+\mathrm{inter}}=\overset{¯}{x}\;{\text{(cold)}}_{\mathrm{intra}+\mathrm{inter}}-\overset{¯}{x}\;{\text{(warm)}}_{\mathrm{intra}+\mathrm{inter}}$$

(6) $$\Delta {\overset{¯}{x}}_{\mathrm{inter}}=\overset{¯}{x}\;{\text{(cold)}}_{\mathrm{inter}}-\overset{¯}{x}\;{\text{(warm)}}_{\mathrm{inter}}$$

(7) $$\Delta {\overset{¯}{x}}_{\mathrm{intra}}=\Delta {\overset{¯}{x}}_{\mathrm{intra}+\mathrm{inter}}-\Delta {\overset{¯}{x}}_{\mathrm{inter}}$$

where Δ*x*_intra+inter_ is the size change attributed to the combined effects of intra‐ and interspecific changes, and Δ*x*_inter_ and Δ*x*_intra_ are the contribution of interspecific and intraspecific changes to Δ*x*_intra+inter_.

In addition to the weighted mean, we also calculated the weighted standard deviation and standard error of the mean. A Welch two‐sample weighted *t*‐test (Bland & Kerry, 1998) was used to assess the difference in the weighted mean between the two periods.

### 2.4 Literature values

Literature values on the diameter range from each species were extracted from the online section of the Modern Dinocyst Key on http://www.marum.de/en/Modern_Dinocyst_Key.html (Table 2; Zonneveld & Pospelova, 2015). For *Spiniferites elongatus* and *S. ramosus*, length and width ranges were converted to diameter ranges by calculating the average of length and width for the minimum and maximum values, respectively.

For individual species, we compared the mean and total size ranges from our measurements to the midpoint (defined as the value in the middle of the literature size range) and size ranges reported in the literature. For the total community, we calculated the weighted mean without intraspecific change (Equation 3) using the literature‐derived midpoints as the species means (Ø_avg_ in Equation 3). This literature based community “mean” was then compared to the community mean obtained from our measurements.

## 3 Results

In both periods, *I. minutum*,* P. dalei*, and *I.? cezare* were the most dominant taxa. However, the relative abundance of these taxa changed between the periods with a relative increase in the abundance of *I. minutum* from about 50% to 65%, and *I.? cezare* from about 4% to 6% and a concomitant decrease in the relative abundance of *P. dalei* from about 20% to 8%, from the warm to the cold period (Figure 2b).

**Figure 5:**
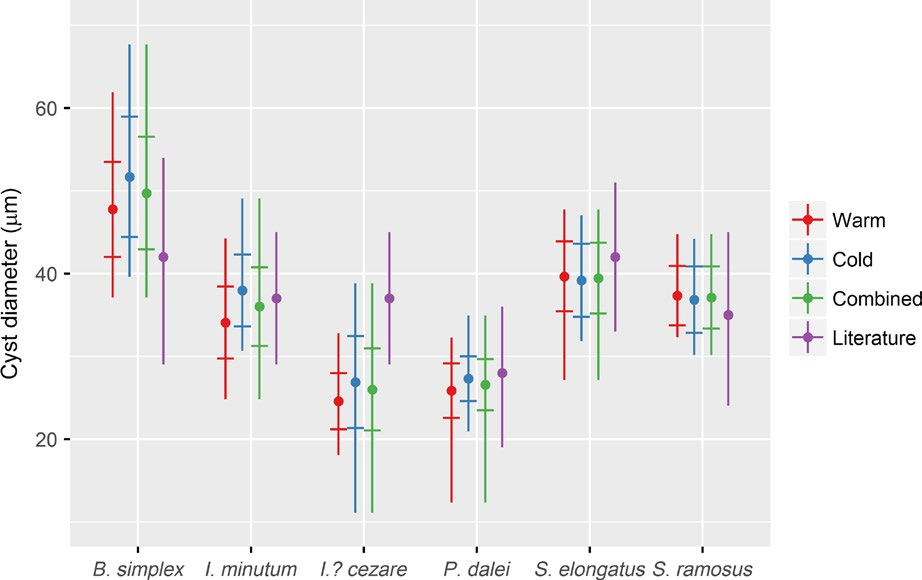
Mean cyst diameter shown by filled dots (•), standard deviation shown by crosshatches (‡), and total size range shown by vertical lines (|) for individual species based on measurements in the warm and cold periods as well as for all observations combined. For the literature values, the point (•) represents the midpoint in the reported range

**Figure 6:**
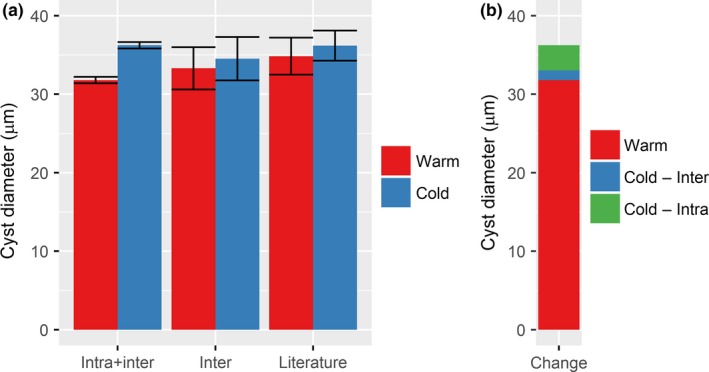
Comparison of the community‐weighted mean diameter (and standard error) between the warm and cold periods and between calculations with and without intraspecific changes as well as from literature‐derived midpoints (a). Change in community‐weighted mean size between warm and cold periods partitioned into intraspecific and interspecific contributions (b)

For the measurements on the species *B. simplex*,* I. minutum*,* I.? cezare*, and *P. dalei* as well as for the community as a whole including intraspecific changes, the mean cyst diameter was significantly larger in the cold period compared with the warm period (Figures 5 and 6; Table 1). For *S. elongatus* and *S. ramosus*, the mean cyst diameters were smaller in the cold compared with the warm period, but these changes were not statistically significant (Figure 5; Table 1). Numerically, size changes were relatively large (Table 1); for example, for *I. minutum*, the mean shifted 3.88 μm from the warm to the cold period (from 34.08 to 37.96), which in linear dimensions is equivalent to an increase of *ca*. 11.4%. Assuming the cysts to be approximately spherical (i.e., *V = r* ^*3*^ × π × *4/3*), the increase in the diameter between the warm and cold periods for *I. minutum* is equivalent to an increase in mean volume of ca. 38%. For the species *B. simplex*,* I.? cezare*, and *P. dalei*, this increase was 27%, 31% and 18%, respectively.

**Table 1:** Summary statistics calculated from cyst diameters and results of *t*‐tests for all species and communities. For communities, the weighted mean, weighted standard deviation, and a weighted *t*‐test are reported

Species/community	Warm period				Cold period			
	Mean (μm)	SD (μm)	SE (μm)	*n*	Mean (μm)	SD (μm)	SE (μm)	*n*
*Brigantedinium simplex*	47.75	5.75	1.05	30	51.68	7.28	1.33	30
*Islandinium minutum*	34.08	4.35	0.60	52	37.96	4.34	0.61	51
*Islandinium? cezare*	24.55	3.39	0.58	34	26.89	5.54	0.75	55
*Pentapharsodinium dalei*	25.85	3.30	0.44	57	27.31	2.71	0.36	55
*Spiniferites elongatus*	39.65	4.22	0.81	27	39.17	4.41	0.96	21
*Spiniferites ramosus*	37.33	3.59	0.82	19	36.84	4.01	0.97	17
Community (intra+inter)	31.80	5.97	0.40	219	36.24	6.20	0.41	229
Community (inter)	33.30	6.60	2.69	6^a^	34.53	6.76	2.76	6^a^
Community (inter; literature)	34.85	5.76	2.35	6^a^	36.19	4.70	1.92	6^a^

Species/community	Δmean (μm)	Δmean (%)	*df*	*p*
*Brigantedinium simplex*	3.93	8.2	55.1	.024
*Islandinium minutum*	3.88	11.4	101.0	<.001
*Islandinium? cezare*	2.34	9.5	87.0	.016
*Pentapharsodinium dalei*	1.46	5.6	107.3	.012
*Spiniferites elongatus*	−0.48	−1.2	42.2	.709
*Spiniferites ramosus*	−0.49	−1.3	32.4	.703
Community (intra+inter)	4.44	14.0	446.0	<.001
Community (inter)	1.22	3.7	9.8	.670
Community (inter; literature)	1.35	3.8	8.8	.562

a At the interspecific level, the number of observations is reduced to the number of species (6) in the calculation of the standard error and in the weighted *t*‐test.

All species showed large variability in cell diameter both in standard deviations and total size ranges (Figure 5; Table 1). Using *I. minutum* in the cold period as an example, the total size range covered about 20 μm with two‐thirds of the observations within about 4 μm from the mean. Thus, even within one standard deviation, the size variability of *I. minutum* in the cold period ranged from 34 to 42 μm which, in terms of volume, means that the largest is about twice the size of the smallest. This variability was consistent between the warm and cold periods across all species. Thus, standard deviations were of comparable size within species between the warm and cold periods showing that, while the mean in most cases was different between the periods, the shape of the size distribution for each species did not change significantly. For *B. simplex*,* I. minutum*, and *P. dalei*, this was also evident in the total size range which showed a shift of the entire size distribution from the warm period to the cold period. *Spiniferites elongatus* and *S. ramosus* had similar ranges and distributions in both periods. The only exception was *I.? cezare* which showed a very large total size range in the cold period which covered the total size range of the warm period.

The change in community mean size from the warm period to the cold period was relatively large with an increase of 4.44 μm (Figure 6a; Table 1). This size change was larger than the change in any of the individual species indicating that, in addition to the changes in size within species, a shift in community composition also contributed to the increase in the community mean between the warm period and the cold period. However, intraspecific change accounted for a much larger fraction of the increase. Specifically, interspecific change accounted for an increase of 1.22 μm, whereas intraspecific change accounted for the remaining 3.22 μm from the warm period to the cold period (Figure 6b; Table 1). When only interspecific changes were taken into account, the difference between the warm and cold periods was, in fact, not significant (Table 1; however, note that the number of observations, and thus degrees of freedom, is much lower than when intraspecific changes are included).

Comparing the measured sizes with literature values did not show a consistent pattern (Figure 5; Table 2). For four of the species (*I. minutum*,* P. dalei*,* S. elongatus*, and *S. ramosus*), the literature midpoints resembled the average of the measurements but, with the exception of *S. ramosus*, the range of measurements extended beyond the reported literature range. For *B. simplex* and *I.? cezare*, both the midpoints and the reported literature size range was noticeably different than the measured values of average and range. The difference between thecommunity mean size in the warm and cold periods was almost the same using literature midpoints as using only interspecific difference from direct measurements (Figure 6a; Table 1), but the community mean was higher for both periods.

**Table 2:** Dinoflagellate cyst sizes extracted from the literature (see http://www.marum.de/en/Modern_Dinocyst_Key.html; Zonneveld & Pospelova, 2015)

Cyst name (vegetative name)	Length (μm)	Width (μm)	Diameter (μm)	Midpoint (μm)
*Brigantedinium simplex (Protoperidinium conicoides)*	–	–	29–54	42
*Islandinium minutum*	–	–	29–45	37
*Islandinium? cezare*	–	–	29–45	37
*Pentapharsodinium dalei*	–	–	19–36	28
*Spiniferites elongatus (Gonyaulax elongata)*	40–59	26–42	33–51	42
*Spiniferites ramosus (Gonyaulax spinifera)*	30–46	17–43	24–45	35

## 4 Discussion

In this study, we have addressed three questions:

### 4.1 Were protists significantly smaller in the warm period compared to the cold period?

Four of the six dinoflagellate cyst taxa studied were smaller in the relatively warm period compared to the relatively cold period. Furthermore, there was a consistent shift in community composition between the two time periods, with a greater dominance of relatively small dinoflagellate taxa in the warm period. Thus, our results show a negative relationship between protist size and temperature. These results support the size–temperature rule (Atkinson, 1994) and indicate that past protist communities follow the same overall temperature–size pattern found in contemporary studies (reviewed in Finkel et al., 2010; Sommer, Peter, Genitsaris, & Moustaka‐Gouni, 2016). Our results therefore provide independent support to studies which, based on contemporary spatial patterns of size and temperature, predict a decrease in protist community mean size in a future warmer ocean (Daufresne et al., 2009; Morán et al., 2010; Winder et al., 2009). The paleoecological approach is thus a viable way of investigating actual temporal size–temperature patterns.

The causal mechanisms linking temperature change to shifts in protist cell size have been widely discussed (López‐Urrutia & Morán, 2015; Marañón, Cermeño, Latasa, & Tadonléké, 2012; Sommer et al., 2016). Currently, there is evidence for both a direct effect and an indirect effect of temperature, because temperature changes affect both metabolic rates as well as nutrient availability in the euphotic zone. In Disko Bay, nutrient concentrations have also been shown to vary with temperature changes (Hansen, Nielsen, Stedmon, & Munk, 2012; Tremblay & Gagnon, 2009) and it is therefore unlikely that the differences in cell size between the warm and cold periods observed in this study can be attributed solely to a direct effect of temperature on size (i.e., 2.5% decrease in cell volume for each 1°C increase; Atkinson et al., 2003). Instead, we hypothesize that the relatively large change in cell size reported here is a result of the combined effects of temperature itself (direct; see Atkinson, 1994; Atkinson et al., 2003) and increased competition for limiting nutrients in the warm period compared with the cold period (indirect; see Kiørboe, 1993).

The two *Spiniferites* taxa (*S. elongatus* and *S. ramosus*) were exceptions to the general pattern in our analysis. Although the slight increases in mean size between warm and cold periods were statistically not significant, it is clear that *S. elongatus* and *S. ramosus* did not decrease in size with increasing temperature. Other studies have also identified protists with apparently contrasting temperature–size relationships although the general trend showed a decrease in size with increasing temperature (Atkinson, 1995; Peter & Sommer, 2012). However, additional analyses are needed to address whether *Spiniferites spp*. indeed constitute such general exceptions.

Changing size is not the only morphometric response to changes in the physical properties of water reported for dinoflagellate cysts. Correlation between spine length and salinity/density has been reported for *Lingulodinium polyedrum*,* Protoceratium reticulatum*, and *Gonyaulax baltica* from controlled laboratory experiments (Ellegaard et al., 2002) and from natural assemblages (Ellegaard, 2000; Mertens et al., 2011) and this relationship has, for example, been used to reconstruct surface salinity in the Black Sea throughout the Holocene (Mertens et al., 2012). As it has been the case with correlations between spine length and salinity, our results show that the relationship between cyst diameter and temperature can potentially be used in paleotemperature reconstructions.

### 4.2 What is the relative importance of intra‐ vs. interspecific change?

By measuring cysts, we were able to detect not only changes in community size structure but also changes in the mean size of individual dinoflagellate taxa. We show that significant intraspecific variations in protist cell size can have a large impact on community size structure. Indeed, a full two‐thirds of the size variation in our study was due to intraspecific variability in cyst size and failing to include this would have led to a significant underestimation of the differences in community mean size between the two periods.

In a recent study, Sommer, Paul, and Moustaka‐Gouni (2015) reported a similar pattern from a mesocosm experiment investigating the effects of temperature and CO_2_ change on phytoplankton size in the Baltic Sea. Here, the contribution of dominant taxa did not change significantly between temperature treatments (9 vs. 15°C), but community mean size was still significantly smaller in the warm treatment compared to the cold treatment due to intraspecific decreases in phytoplankton cell size with increased temperature. For terrestrial plants, Siefert et al. (2015) explored the relative extent of intraspecific trait variation compared with interspecific trait variation in a global meta‐analysis of plant communities and found that about 30% of the variation within and among communities was due to intraspecific variation. Furthermore, the result depended on the trait type, with the contribution of intraspecific variation increasing for whole‐organism traits like size.

The relative importance of intra‐ and interspecific variation in driving changes in protist cell size will probably differ between different systems and species assemblages. Recently, Sommer et al. (2016) reviewed the literature on the temperature–size rule in phytoplankton. They assessed the current evidence on the importance of intraspecific size variability in driving community size patterns and found that intraspecific values had been reported in only a handful of community‐level studies thus far. While the review reported a negative relationship with increasing temperature in both intra‐ and interspecific sizes, the authors concluded that interspecific changes in general are larger than intraspecific changes. Our study demonstrates that this pattern does not always hold and that intraspecific size change can be more important than shifts in species composition in driving community‐level size patterns. By ignoring the role of within‐species variability, we risk misinterpreting protist size as well as other trait patterns.

### 4.3 Are literature values of species size adequate to describe local size distributions and changes in community size?

It is clear from our study that collated literature size ranges and midpoint values do not necessarily represent local dinoflagellate cyst sizes. Thus, the size range of five of six species in our study extended beyond the reported literature size range and two species showed mean sizes that were very different from the literature midpoint. *Islandinium? cezare* represented the most obvious discrepancy with both the size range and midpoint being significantly smaller in this study, when compared to literature values. A possible explanation for this difference is that *Islandinium? cezare* is an Arctic species. According to the reported distribution of *I.? cezare* cysts (Zonneveld et al., 2013), Disko Bay is located at the warm end of *I.? cezare*'s temperature range, and given the temperature–size rule, indeed it is expected that *I.? cezare* would be smaller in this area than reported literature values. While some species showed a better match between measured and literature‐derived values, the important point here is that it is impossible a priori to determine whether literature values are representative because the full size range and also the phenotypic response to environmental change are usually unknown.

Changes in community mean size resulting from intraspecific change were larger when calculated from literature midpoints compared to the actual measurements. This difference was primarily caused by *I.? cezare*, but the two other primary dominating species (*I. minutum* and *P. dalei*) also exhibited slightly higher literature midpoints compared with the measured mean. Of these, *I. minutum* is also adapted to cold waters (Head, Harland, & Matthiessen, 2001; Zonneveld et al., 2013) and it is possible that this taxon has also been collected at the warmer end of its temperature range. This can possibly explain why the observed community mean was lower than the “mean” derived from the literature. *Pentaphasodinium dalei* on the other hand is a temperate‐cold water species found further south than Disko Bay but not in high numbers further north and we would therefore have expected measurements of this taxa to be smaller than the literature midpoint. Unfortunately, it is not possible in this study to investigate the causes of this discrepancy. It is, however, clear that the community mean reflects the size estimates of the most dominant taxa and that any biases in these estimates will have a large impact on the resulting community estimate. Therefore, as in the case of individual species, literature values cannot be expected to provide numerically accurate estimates of community mean size.

Despite these differences, changes in community mean size between the warm and cold periods were actually of very similar magnitudes when interspecific changes were calculated from literature values and compared to the interspecific community mean size based on measured means. Thus, even if the literature based community mean was considerably different from the real community mean size, it still appeared to capture the direction of the relationship between protist size and temperature. It therefore seems fair to ask under which circumstances interspecific estimates can be used and which types of questions can be answered?

In the literature on terrestrial biota, it has been indicated that the importance of intraspecific change decreases with increasing scale (Siefert et al., 2015). In this context, even if the magnitude of change is likely underestimated, our results support that it may be possible to investigate general patterns, that is, relative differences, in protist community mean size using interspecific size estimates at large spatial scales, for example, at the global scale (Chen et al., 2011) and/or at large temporal scales, for example, millennial or geological timescales (Finkel et al., 2005, 2007).

However, it has also been suggested that the relative importance of intra‐ vs. interspecific variation depends on the trait and the study system (Albert et al., 2010). For example, for higher plants it has been shown that even a small amount of intraspecific variation can have a large effect on ecosystem processes at the community level (Jung et al., 2010). This could be especially important for protists because protist size is strongly linked to ecosystems functioning through its effects on biogeochemical cycling, energy fluxes, and export production (Hilligsøe et al., 2011; Yvon‐Durocher & Allen, 2012). In relatively small‐scale studies, for example, local studies at up to centennial timescales, and/or in studies where the magnitude of change is important, intraspecific changes are likely necessary to accurately investigate changes in protist community mean size.

## 5 Conclusion

In this study, we investigated differences in marine protist mean size at species and community levels between two different climate periods by analyzing samples from a sediment record collected in Disko Bay, Greenland. Measurements were performed on six of the most abundant dinoflagellate cyst species representing about 80% of the total abundance. We conclude that: (1) The marine protist community showed an inverse relationship between mean size and temperature with smaller cyst sizes at both the intra‐ and interspecific levels during the period of relatively warm temperatures, compared with the period of relatively cold temperatures; (2) changes at the species level (intraspecific) were responsible for more than 70% of the shift in community mean size, whereas species shifts (interspecific) explained less than 30% of the observed change; (3) literature values on size ranges and midpoints for individual taxa were in most cases not adequate to describe local size distributions; (4) depending on the spatiotemporal scale and the study question, interspecific‐level estimation may be adequate to investigate general patterns in protist community mean size. We thus confirm the great potential for using the fossil record to test hypotheses of responses of both individual species and communities to environmental change and recommend that the important role of within‐species variability is included in future investigations of environmental change impacts on community‐level patterns.

## Conflict of Interest

None declared.
